# Lack of pharmacokinetic interaction between derazantinib and naringin in rats

**DOI:** 10.1080/13880209.2023.2185641

**Published:** 2023-03-08

**Authors:** Ya-nan Liu, Jie Chen, Xinhao Xu, Yingying Hu, Jin-yu Hu, Ren-ai Xu, Guanyang Lin

**Affiliations:** a Department of Pharmacy, The First Affiliated Hospital of Wenzhou Medical University, Zhejiang, China; b School of Pharmaceutical Sciences, Wenzhou Medical University, Zhejiang, China

**Keywords:** Intrahepatic cholangiocarcinoma, dose adjustment, pharmacokinetics, UPLC-MS/MS

## Abstract

**Context:**

Derazantinib—an orally bioavailable, ATP competitive, multikinase inhibitor—has strong activity against fibroblast growth factor receptors (FGFR)2, FGFR1, and FGFR3 kinases. It has preliminary antitumor activity in patients with unresectable or metastatic FGFR2 fusion-positive intrahepatic cholangiocarcinoma (iCCA).

**Objective:**

This experiment validates a novel sensitive and rapid method for the determination of derazantinib concentration in rat plasma by ultra-performance liquid chromatography tandem mass spectrometry (UPLC-MS/MS), and applies it to the study of drug-drug interaction between derazantinib and naringin *in vivo*.

**Materials and methods:**

A Xevo TQ-S triple quadrupole tandem mass spectrometer was used for mass spectrometry monitoring in selective reaction monitoring (SRM) mode with transitions of *m/z* 468 96 → 382.00 for derazantinib and *m/z* 488.01 → 400.98 for pemigatinib, respectively. The pharmacokinetics of derazantinib (30 mg/kg) was investigated in Sprague-Dawley (SD) rats divided into two groups (with the oral pretreatment of 50 mg/kg naringin or not).

**Results:**

The newly optimized UPLC-MS/MS method was suitable for the determination of derazantinib in rat plasma. It was also successfully employed to evaluate the effect of naringin on derazantinib metabolism in rats. After pretreatment with naringin, there was no significant difference in the pharmacokinetic parameters (AUC_0→t_, AUC_0→∞_, t_1/2_, CLz/F, and C_max_) of derazantinib when compared with derazantinib alone.

**Conclusion:**

Co-administration of naringin with derazantinib was not associated with significant changes in pharmacokinetic parameters. Thus, this study suggests that the combination of derazantinib with naringin can safely be administered concomitantly without dose adjustment.

## Introduction

Following hepatocellular carcinoma (HCC), cholangiocarcinoma (CCA) is the second most common liver malignancy, which was classified as intrahepatic or extrahepatic (perihilar and distal) based on the tumor location in the biliary tract (Rizvi and Gores 2013; Braun et al. 2021; King and Javle 2021). As a rare but aggressive and fatal subtype of CCA, intrahepatic cholangiocarcinomas (iCCA) has shown an increasing trend in incidence and mortality worldwide in recent decades (Gourd 2019; Aitcheson et al. 2021). It was found that 30–40% of iCCA patients had potential targeted aberrations after the whole gene map sequencing. The most common was the fusion/rearrangement of fibroblast growth factor receptors (FGFR)2, and the fusion only occurred in iCCA (Jain et al. 2018; Lowery et al. 2018; Mazzaferro et al. 2019; Braun et al. 2021; Silverman et al. 2021). Therefore, FGFR2 has great potential in targeted treatment of iCCA, and FGFR2 inhibitors are also used as the treatment of iCCA (Abou-Alfa et al. 2020).

**Figure 1.**
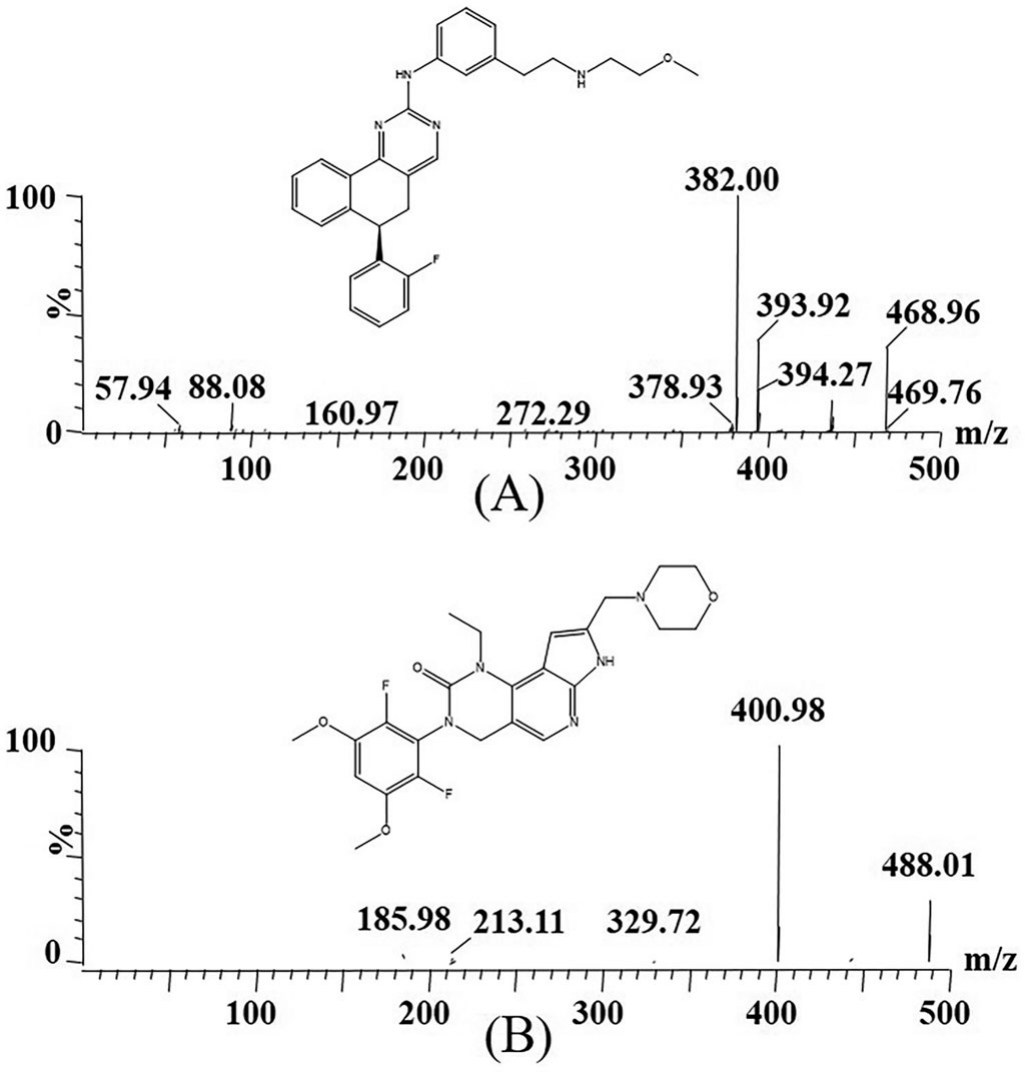
Mass spectras of derazantinib (A) and pemigatinib (IS, B) in this study.

As an orally bioavailable, ATP competitive pan-FGFR inhibitor, derazantinib (Figure 1(A)) has strong activity against FGFR1, FGFR2, and FGFR3 kinases (Hall et al. 2016). It is dose-dependent in the iCCA cell line, which can inhibit cell growth and increase apoptosis, and reduce signal transduction in the mitogen activated protein kinase (MAPK) pathway (Raggi et al. 2019). In addition, derazantinib has preliminary antitumor activity in patients with unresectable or metastatic FGFR2 fusion-positive iCCA (Gourd 2019). The clinical trial application (CTA) of derazantinib in China was officially approved by the end of April 2019 and is currently being developed for the treatment of other tumor types with a high frequency of FGFR mutations as monotherapy and in combination with either immunotherapy or chemotherapy (Raggi et al. 2019; Kelley et al. 2020; Lacouture et al. 2021). As a targeted drug for the treatment of iCCA, derazantinib is a promising drug for iCCA patients with FGFR2 gene abnormalities after previous chemotherapy (Papadopoulos et al. 2017).

Naringenin, of the flavonoid family, can increase the exposure of related antigens, enhance cytotoxicity as well as promote immunity when combined with anticancer drugs (Memariani et al. 2021). It also can regulate the level of T lymphocytes in the body and has the potential to develop into an immune regulator (Elsawy et al. 2020). In the treatment of liver cancer, naringin can induce apoptosis of human hepatocellular carcinoma HepG2 cells, inhibit the invasion and metastasis of HCC by inhibiting a variety of signal transduction pathways according to the existing research data (Yen et al. 2015; Banjerdpongchai et al. 2016; Chtourou et al. 2016). Thus, naringin has a certain medical potential for the treatment of iCCA. Moreover, the first-line treatment for iCCA is gemcitabine-cisplatin chemotherapy, which will inevitably cause renal injury, while naringin can treat this renal injury to a certain extent (Townsend et al. 2003; Miller et al. 2010; Chtourou et al. 2016; Kelley et al. 2020).

Derazantinib has activity against vascular endothelial growth factor receptor-2 (VEGFR2), which can significantly reduce tissue permeability and tumor function of vascular system (Papadopoulos et al. 2017; Braun et al. 2021). In addition, naringin can inhibit the production of vascular endothelial growth factor (VEGF) (Memariani et al. 2021). Therefore, the combination of naringin and derazantinib after chemotherapy has great potential medical treatment (Memariani et al. 2021). It is of great significance and practical value to explore whether there is drug-drug interaction or mutual inhibition between them. The invention and establishment of quantitative analysis method of derazantinib in plasma is the necessary condition, and allow to study its pharmacokinetic characteristics for assessing whether naringin has inhibitory effect on derazantinib metabolism.

Thus, in this experiment, our purpose was to invent and validate a sensitive and rapid method for the determination of derazantinib concentration in rat plasma by ultra-performance liquid chromatography tandem mass spectrometry (UPLC-MS/MS). The novel established UPLC-MS/MS technique was suitable for the pharmacokinetics and drug-drug interaction study of derazantinib in rats.

## Materials and methods

### Chemical materials and reagents

Derazantinib was purchased in powder form from Shanghai Chuangsai Technology Co., Ltd. (Shanghai, China), and pemigatinib (used as internal standard, IS, Figure 1(B)) was also provided by it. High performance liquid chromatography (HPLC) grade methanol and acetonitrile were supplied by Merck company (Darmstadt, Germany), and HPLC grade formic acid was provided by Anaqua Chemicals Supply (ACS, American). The ultrapure water was prepared using a Milli-Q Water Purification System (Millipore, Bedford, MA, USA).

### Animal experiments

Twelve Sprague-Dawley (SD) rats (male, body weight 200 ± 20 g) were provided by the Experimental Animal Center of The First Affiliated Hospital of Wenzhou Medical University (Zhejiang, China). The rats were fed with unlimited feed and water for one week under standard feeding conditions (temperature 25–28 °C, humidity 50–60%, 12 h light/dark). According to the Regulations for The Care and Use of Experimental Animals, all the behaviors and operations of this experiment had been approved by the institutional Ethics Committee of The First Affiliated Hospital of Wenzhou Medical University (Approval No: WYYY-AEC-2022-028).

All rats were randomly divided into two groups (*n* = 6): control (group A) and experimental (group B). Before the experiment, 12 rats were fasted for 12 h without limiting drinking water. Naringin in group B was administered by gavage with the dose of 50 mg/kg, dissolved in 0.5% sodium carboxymethyl cellulose (CMC-Na) solution, while the same amount of CMC-Na solution was given to rats at the same time for group A. After the last administration of 30 min, group A and B were given of derazantinib (dissolved in CMC-Na solution) by gavage according to the ratio of 1 kg to 30 mg. Then, 1.5 mL Eppendorf (EP) tubes with heparin in advance were prepared, and blood were taken from the caudal vein according to the following time points: 0.333, 0.667, 1, 1.5, 2, 3, 4, 6, 8, 12, 24, and 48 h. The obtained blood samples were placed into EP tubes before centrifugated at 13000 rpm at 4 °C for 10 min. Then, 100 µL supernatant was taken and stored at −80 °C for the next step of analysis, with an appropriate and simple UPLC-MS/MS method.

### Instrumentations and analytical conditions

A Waters Xevo TQ-S triple quadrupole tandem mass spectrometer (Milford, MA, USA) and a Waters ACQUITY UPLC I-Class system (Milford, MA, USA) constituted this UPLC-MS/MS system, which used electrospray ionization (ESI) source. In the experiment, 0.1% formic acid solution and acetonitrile were used as the mobile phase in the instrument, and the model of the column was ACQUITY UPLC BEH C18 column (2.1 mm × 50 mm, 1.7 μm). A gradient elution was used to separate derazantinib and IS, and the linear gradient elution procedure was as follows: 0–0.5 min (10% acetonitrile was eluted at a flow rate of 0.30 mL/min); 0.5–1.0 min (acetonitrile, 10–90%); 1.0–1.4 min (acetonitrile, 90%) and 1.4–1.5 min (acetonitrile, 90–10%). The single injection volume was 2 μL, and the single analysis time was 2.0 min. In addition, the injection temperature was 10 °C, and the column temperature was 40 °C.

A Xevo TQ-S triple quadrupole tandem mass spectrometer was used for mass spectrometry monitoring, combined with an ACQUITY UPLC system for analysis. In positive ion mode, the collision energy of derazantinib and IS were 25 eV and 15 eV, respectively, and the cone voltage were 20 V and 30 V, respectively. In addition, the selective reaction monitoring (SRM) modes of ion transitions of derazantinib and IS were *m/z* 468.96 → 382.00 and *m/z* 488.01 → 400.98, respectively.

### Standard solutions, calibration curves and quality control (QC) samples

Firstly, methanol was used to prepare the stock solution of derazantinib and IS with the respective concentration of 1.00 mg/mL, and they were stored in the refrigerator at −80 °C before they were employed to prepare for the quality control (QC) samples and calibration curves. The range of the standard curve (0.5–1000 ng/mL) should be determined according to the plasma drug concentration measured in the animal experiment, and should cover all the concentrations as far as possible. A series of working solutions (5–10000 ng/mL) were obtained by using methanol for gradient dilution, and different concentration points on the standard curve were prepared by 10-fold dilution with blank plasma. The final concentrations of QC samples were 1 (LQC), 80 (MQC) and 800 (HQC) ng/mL, and the working solution configuration of each concentration was similar to the standard curve. In addition, the concentration of IS was 200 ng/mL, which was obtained by diluting the stock solution with methanol.

### Sample preparation

When preparing the sample, the plasma sample was treated by protein precipitation method, and acetonitrile was used as the precipitant. IS working solution (10 μL) was placed into the 1.5 mL EP tube containing 100 μL plasma sample, and 300 μL acetonitrile was added and fully vortexed for 1.0 min. The EP tubes were put into a centrifuge for centrifugation (rotating speed 13000 rpm, temperature 4 °C, time 10 min), and then 100 μL supernatant was taken into the injection bottle before analysis.

### Method validation

The verification method and validation procedures were accordance with Food and Drug Administration (FDA) guidelines for the validation of tests, including the calibration curve, stability, matrix effect, recovery, lower limit of quantification (LLOQ), precision and accuracy (Booth et al. 2015).

In order to verify the absence of endogenous interference near the retention times of derazantinib and IS and the selectivity of the analytical method, we investigated and evaluated three type samples of blank rat plasma, spiked plasma (at LLOQ concentration) and real rat plasma after oral administration of derazantinib. The sensitivity of the method was reflected by LLOQ, which was also the lowest quantitative point, and its signal-to-noise (S/N) ratio was ≥ 10. According to the relationship between the peak area ratio (y, analyte/IS) and the nominal concentration (x), a standard curve was constructed to calculate the drug concentration of the sample with a weighting factor of the reciprocal of concentration (1/*x*^2^).

Six plasma samples of the same concentration were detected at each level and used in the calculation of precision, accuracy, recovery and matrix effect of derazantinib. In addition, QC samples of three different concentrations (including 1, 80 and 800 ng/mL) and LLOQ (0.5 ng/mL) were repeatedly detected for three consecutive days for inter-day precision and accuracy evaluation. The recovery of the analyte was calculated by comparing the concentration of the analyte in the matrix before and after extraction. The matrix effect was calculated by comparing the peak area of spiked samples after extraction with the corresponding results of pure solution.

The stability was also evaluated after 3 h at room temperature (short term) and 3 weeks at −80 °C (long term). In addition, the stability for 4 h after complete sample preparation and the stability of three complete freeze-thaw (–80 °C to room temperature) were tested and evaluated in the sampler at 10 °C. The evaluation of recovery, matrix effect and stability of derazantinib included three different levels of 1, 80, and 800 ng/mL.

## Results and discussion

### Method design and optimization

In this experiment, we optimized the parameters of MS in order to achieve better analysis of derazantinib and IS and obtain more accurate analysis results. The mobile phase used in this method was composed of 0.1% formic acid aqueous solution and acetonitrile. The optimized gradient elution procedure can make the analytes of derazantinib and IS have good peak and separation effect within 2 min. The retention times of the analyte and IS were 1.53 min and 1.35 min, respectively, and the quantitative fragment ions were *m/z* 468.96 → 382.00 and *m/z* 488.01 → 400.98, respectively.

Plasma samples need to be processed before UPLC-MS/MS detection in order to reduce the interference of endogenous substances, especially proteins. In this study, we used a simpler protein precipitation method to extract plasma. Acetonitrile was chosen as the precipitant, which could obtain the allowable recovery and matrix effect.

### Method validation

#### Selectivity

**Figure 2.**
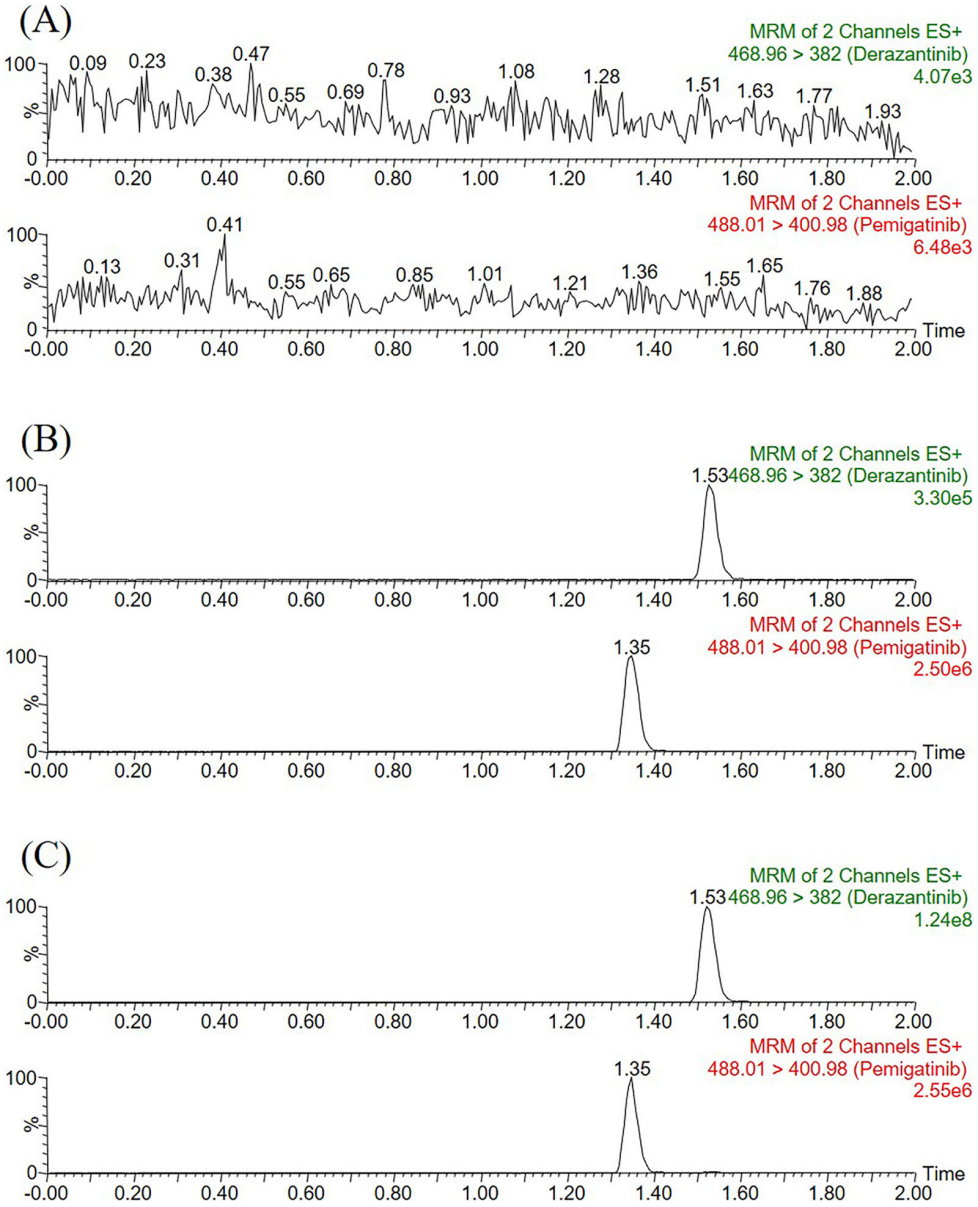
Representative chromatograms of derazantinib and IS in rat plasma: (A) blank plasma; (B) blank plasma spiked with standard solution at LLOQ (0.5 ng/mL) and IS; (C) sample obtained from a rat after oral administration of 30 mg/kg derazantinib.

As Figure 2 displayed, the sample of pretreated rat blank plasma was less than 20% of the analyte LLOQ response and less than 5% of the IS response. The retention times of derazantinib and IS were 1.53 min and 1.35 min, respectively. Substance that may significantly interfere with the detection of them was not found in the chromatogram, and the peak of the real sample could also be accurately detected in the plasma obtained after intragastric administration. Therefore, the optimized method developed in this study had good selectivity and specificity for the detection of derazantinib and IS in rat plasma.

### Linearity and sensitivity

The concentration range was 0.5–1000 ng/mL of the standard curve, and each point of the standard curve was obtained by gradient dilution of the corresponding working solution. The linear regression equation fitted by the least square method was *y* = 0.00683 × *X* + 0.00259 (*r*^2^ = 0.9993), which had a good linear relationship and was met the requirements of methodological detection. The sensitivity of the method was measured by LLOQ with the value of 0.5 ng/mL, and the precision and accuracy were less than 20%.

### Precision, accuracy, recovery and matrix effect

Intra-day and intra-day precision and accuracy were measured from the four concentration levels of LLOQ, LQC, MQC as well as HQC. In this specific detection method, four concentrations of working solution were obtained by gradient dilution of 1.0 mg/mL stock solution of derazantinib. Six samples (*n* = 6) were made for each concentration, which were tested horizontally for three consecutive days. The experimental results were displayed in Table 1, which showed that the UPLC-MS/MS method had excellent precision and accuracy.

**Table 1.** The precision and accuracy of derazantinib in rat plasma (*n* = 6).

Analyte	Concentration (ng/mL)	Intra-day		Inter-day	
		RSD%	RE%	RSD%	RE%
Derazantinib	0.5	9.7	12.3	13.9	6.4
	1	8.5	14.4	10.3	4.8
	80	7.6	13.8	6.2	9.4
	800	2.6	–2.4	4.9	–6.8

The detection of recovery and matrix effect was basically consistent with the above experimental methods of precision and accuracy, but there was one difference. The recovery and matrix effect only need to be measured at the concentration levels of LQC, MQC and HQC. Under the treatment of this method, no endogenous impurities affecting the determination of the analyte and IS in plasma were observed in the experiment. The calculation results of recovery and matrix effect of three concentration levels were shown in Table 2, indicating that this method was reliable and effective.

**Table 2.** Recovery and matrix effect of derazantinib in rat plasma (*n* = 6).

Analyte	Concentration Added (ng/mL)	Recovery (%)		Matrix effect (%)	
		Mean ± SD	RSD (%)	Mean ± SD	RSD (%)
Derazantinib	1	86.1 ± 10.2	11.8	98.4 ± 10.5	10.6
	80	88.8 ± 7.4	8.3	114.0 ± 7.8	6.8
	800	89.6 ± 3.5	3.9	108.7 ± 2.9	2.7

### Stability

The investigation of stability was carried out under different experimental conditions with three concentration gradients of LQC, MQC and HQC, including the stability of the extracted samples after being placed in the injector for 4 h, the stability of blank plasma after adding derazantinib at room temperature for 3 h, repeated freezing and thawing from −80 °C to room temperature for 3 times, and the stability after being placed at −80 °C for at least 3 weeks. In the above stability experiments, the experimental results (Table 3) met the detection standards of the methodology, which proved that derazantinib was stable and reliable in conventional experiments.

### Animal study

**Figure 3.**
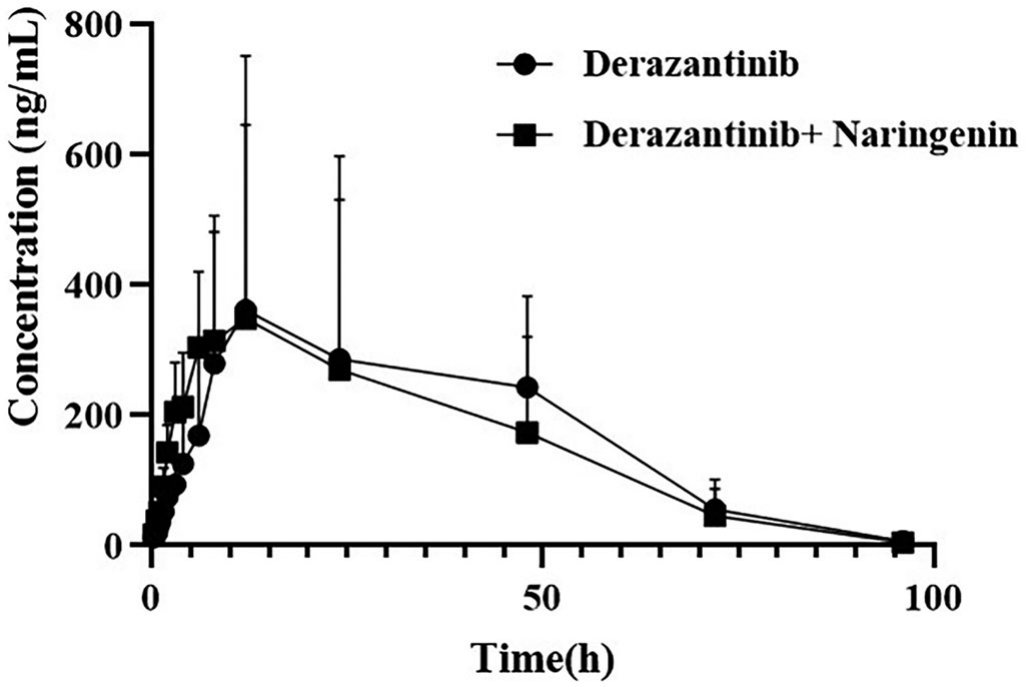
Mean plasma concentration time curve of derazantinib after oral administration of a single dose of 30 mg/kg in rats alone or combination with naringin (*n* = 6).

Table 4 showed the main pharmacokinetic results obtained from noncompartment model analysis with or without naringin in rats. Figure 3 showed the mean plasma concentration time curve of derazantinib alone and combination with naringin in rats. From the results of pharmacokinetic parameters, it could be seen that the maximum plasma concentration (C_max_) of derazantinib in a single oral administration reached 637.174 ± 85.905 ng/mL, which was close to the previously reported paper (Papadopoulos et al. 2017). After analysis, it was found that there was no significant difference in the pharmacokinetic parameters of derazantinib between the two groups. There are likely to be a number of reasons for the pharmacokinetic findings of the current study. It was reported that CYP2C9 is the main metabolic enzyme of naringin, which can inhibit CYP3A1/2 metabolism and P-glycoprotein (P-gp) transport in rats and cause drug interaction (Lim and Choi 2006; Bai et al. 2020). In addition, *in vitro*, derazantinib mainly interacts with plasma due to its strong binding force, and is mainly metabolized by CYP3A4 (Braun et al. 2021). The lack of pharmacokinetic interaction between derazantinib and co-administered naringin, do not share the same elimination pathways. CYP3A4 is only one of a number of pathways by which derazantinib is degraded, whereas it does not appear to be the primary pathway for naringin metabolism. In addition, there is no transporter that commonly affects the pharmacokinetic variables of derazantinib. Thus, co-administered derazantinib and naringin did not result in a significant pharmacokinetic drug interaction, as suggested by the results of our study in rats. However, further experiments are needed to confirm this result.

**Table 3.** Stability results of derazantinib in plasma under different conditions (*n* = 6).

Analyte	Concentration added (ng/mL)	Room temperature, 3 h		Autosampler 10 °C, 4 h		Three freeze-thaw		–80 °C, 3 weeks	
		RSD (%)	RE (%)	RSD (%)	RE (%)	RSD (%)	RE (%)	RSD (%)	RE (%)
Derazantinib	1	6.9	13.5	7.7	11.3	6.9	13.7	6.4	13.2
	80	4.4	1.0	3.2	–2.2	6.2	10.2	3.3	13.2
	800	2.5	7.2	2.2	6.6	4.0	–0.2	2.7	0.7

**Table 4.** The main pharmacokinetic parameters of derazantinib with or without naringenin in rats (*n* = 6, Mean ± SD).

Parameters	Derazantinib	Derazantinib + Naringenin
AUC_0→t_ (ng/mL•h)	26226.389 ± 5571.040	25035.563 ± 1253.775
AUC_0→∞_ (ng/mL•h)	26969.851 ± 5248.942	25434.822 ± 1673.341
MRT_0→t_ (h)	31.521 ± 2.848	29.887 ± 3.565
MRT_0→∞_ (h)	31.858 ± 2.818	30.169 ± 3.630
t_1/2_ (h)	17.056 ± 6.075	13.058 ± 4.540
T_max_ (h)	12 ± 0	15 ± 7.348
CLz/F (L/h/kg)	1.148 ± 0.220	1.184 ± 0.072
Vz/F (L/kg)	29.060 ± 12.727	22.013 ± 6.456
C_max_ (ng/mL)	637.174 ± 85.905	620.412 ± 99.941

## Conclusion

After the progress of first-line systemic chemotherapy of iCCA, derazantinib monotherapy showed good safety and good antitumor activity in the population of patients with iCCA, with a daily dose of 300 mg. Naringin, as a kind of traditional Chinese medicine component that can promote immunosuppression and repair renal injury, has great medical potential in combination with derazantinib. This reminds us that in the treatment of difficult and miscellaneous diseases, we could pay more attention to the role and application of Chinese herbal medicine and its main components in the first-line clinic, and combined medication is a good choice. In this study, we firstly established a reliable, rapid and robust UPLC-MS/MS method to quantify the concentration of derazantinib in rat plasma. At the same time, UPLC-MS/MS method was successfully applied to the pharmacokinetics of derazantinib with or without potent antitumor agent naringin in rats. And, the results displayed that there was no drug-drug interaction between naringin and derazantinib in rats.
